# Genome-wide association study of gestation length in German Holsteins

**DOI:** 10.5194/aab-69-563-2026

**Published:** 2026-10-06

**Authors:** Maximilian Zölch, Valentin P. Haas, Christin Schmidtmann, Hatem Alkhoder, Jens Tetens, Jörn Bennewitz

**Affiliations:** 1 Institute of Animal Science, University of Hohenheim, 70599 Stuttgart, Germany; 2 IT Solutions for Animal Production (vit), 27283 Verden, Germany; 3 Department of Animal Sciences, University of Goettingen, 37077, Göttingen, Germany

## Abstract

Gestation length (GL) is important in seasonal calving systems and in the calving trait complex, because it affects traits such as stillbirth (SB) and calving ease. Utilizing imputed high-density genotypes from 34 497 German Holstein cows, this study aimed to identify genomic regions associated with GL. For this, a genome-wide association study (GWAS) was conducted, and the results were interpreted by searching for previously reported adjacent quantitative trait loci (QTLs).

Four GL traits were analyzed: the direct calf effects on GL in first (GL_d1) and second (GL_d2) parity, along with the maternal effects on GL in first (GL_m1) and second parity (GL_m2). GWAS of direct effects on GL resulted in significant variants on BTA14, 18, and 19 for GL_d1, and on BTA18 and BTA19 for GL_d2. Regarding the maternal effect, only one variant on BTA19 was significant in the second parity. Adjacent to significant variants were known QTLs for several reproduction traits including SB.

Overall, the study provides insights into the complex genetic architecture of GL in German Holstein cattle. The identified genomic regions are supported by previous studies and thereby strengthen the basis for future research.

## 1 Introduction

Gestation length (GL) is a relevant aspect of fertility in dairy cows and is related to traits such as calving ease, dystocia, and stillbirth (SB) (Stachowicz et al., 2023). GL is characterized by an intermediate optimum, as shortened GL reduces calf viability, whereas overly prolonged GL increases birth weight and consequently the risk of calving difficulties (Vieira-Neto et al., 2017). Measured by the risk of perinatal mortality, an overly shortened GL is of higher relevance (Jourdain et al., 2024). Zölch et al. (2026a) have discovered a significant global and local genetic correlation between GL and SB. Consequently, a better understanding of the genetic architecture behind GL could support the breeding efforts for decreasing SB rates by utilizing locally segmented correlations pointing in opposite directions. Genome-wide association studies (GWASs) on GL have been performed, among others, for the Holstein population in Canada (Aponte et al., 2024), France (Jourdain et al., 2024), and Ireland (Purfield et al., 2019). However, these studies followed a window-based approach or were limited by lower marker density (50 K) or the sole inclusion of sires, preventing the analysis of maternal effects. To our knowledge, using imputed high-density (HD) genotypes, no single-marker GWAS for direct and maternal effects on GL has been published.

Therefore, our study aimed to improve the understanding of the genetic architecture behind the direct and maternal effects on GL in German Holsteins. To this end, we conducted a single-marker GWAS to identify trait-associated single nucleotide polymorphisms (SNPs) and then linked these variants to known quantitative trait loci (QTLs).

## 2 Materials and methods

### 2.1 Data description

The study included 34 497 German Holstein dairy cows, initially selected by Schneider et al. (2023). Their genotypic data were provided by Vereinigte Informationssysteme Tierhaltung w.V. (VIT) (Verden, Germany) based on 50 K or low-density SNP chips with a subsequent imputation to the 50 k level (Segelke et al., 2012). All genotype preparation was performed with PLINK (version 2.00a5.12; Chang et al., 2015). SNPs with a minor allele frequency (MAF) <0.01 were removed from the 50 K dataset, reducing the number of available SNPs from 44 747 to 44 126. Based on 1278 Holstein cows genotyped for 585 517 markers, Križanac et al. (2025) further imputed these genotypes to HD using BEAGLE version 5.1 for phasing and BEAGLE version 5.2 for the imputation itself (Browning and Browning, 2007; Browning et al., 2018). The resulting HD genotypes were filtered for MAF < 0.03 and deviation from the Hardy–Weinberg equilibrium ( $P<1\times 10^{-8}$ ), resulting in 479 759 remaining SNPs. The SNP base pair positions follow the ARS-UCD1.2 reference genome assembly.

Direct and maternal GL breeding values were estimated simultaneously by VIT using a single-step SNP BLUP implemented in MiX99 (MiX99 Development Team, 2024). The underlying variance components were obtained from the study by Haile-Mariam and Pryce (2019), as estimates based on German Holsteins were not available. The subsequent deregression followed the method described in Liu and Masuda (2021). The deregressed proofs (DRPs) differentiate between the calf's direct effect on GL in the first (GL_d1) and second gestation (GL_d2), as well as the cow's maternal effect in the first (GL_m1) and second gestation (GL_m2). The trait-specific number of observations was 12 658 for GL_d1, 6141 for GL_d2, 31 007 for GL_m1, and 25 112 for GL_m2.

### 2.2 Statistical analysis

RStudio (version 2025.5.0.496) with R (version 4.4.0) (R Core Team, 2024; Posit team, 2025) was used for data processing and visualization.

Single-marker GWAS was conducted in GCTA (version 1.94.1; Yang et al., 2011) for the four GL traits using the following mixed linear model:

1 y=1μ+bx+Zg+e,

with vector y containing the DRPs for GL_d1, GL_d2, GL_m1, or GL_m2. 1 µ represents the vector of 1 multiplied by the intercept μ . The b denotes the fixed additive genetic effect of the candidate SNP to be tested for association, and x incorporates the genotype vector coded as 0, 1, 2. The vector g denotes the random polygenic effect with an assumed distribution of $g∼N(0,G_{\mathrm{HD}}\sigma_{g}^{2})$ . The HD genotypes were used both for constructing the genetic relationship matrix (GRM) $G_{\mathrm{HD}}$ following the method of Yang et al. (2010) and the GWAS. $\sigma_{g}^{2}$ denotes the additive genetic variance, and Z is an incidence matrix. The e is the residual with a distribution of $e∼N(0,I\sigma_{e}^{2})$ . I represents an identity matrix, and $\sigma_{e}^{2}$ is the residual variance.

To evaluate the extent of false-positive inflation in the GWAS results, the genomic inflation factor $\lambda_{\mathrm{GC}}$ was calculated as the ratio of the median of the observed chi-square test statistics to the expected median (van den Berg et al., 2019). Two significance thresholds were applied: a Bonferroni‐corrected genome-wide significance threshold of $p\leq \frac{0.05}{\text{number of SNPs}}$ and a suggestive threshold of $p\leq 5\times 10^{-5}$ . The GWAS results were visualized as Manhattan plots with the R package qqman (Turner, 2018). The R package GALLO was applied to the genomic regions ±25 kb around genome-wide significant SNPs to annotate the results with known QTL (Fonseca et al., 2020). GALLO utilized the Cattle QTL Database release 56 as a source for the QTL (Hu et al., 2023).

## 3 Results

The Manhattan plots of the GL traits are shown in Figs. 1 and 2. The GWAS on GL resulted in Bonferroni-significant variants for all GL traits, except GL_m1. Associations for GL_d2 exist on BTA18 (one variant) and 19 (41 variants). For GL_d1, the same associations were observed plus three variants on BTA14, 15 more on BTA18, and 29 more on BTA19. Only one variant on BTA19 was significantly associated with GL_m2. A list of all Bonferroni-significant variants is provided in Table S1 in the Supplement (Zölch et al., 2026b). The inflation factor $\lambda_{\mathrm{GC}}$ was close to 1 for all GL traits (not shown).

**Figure 1:**
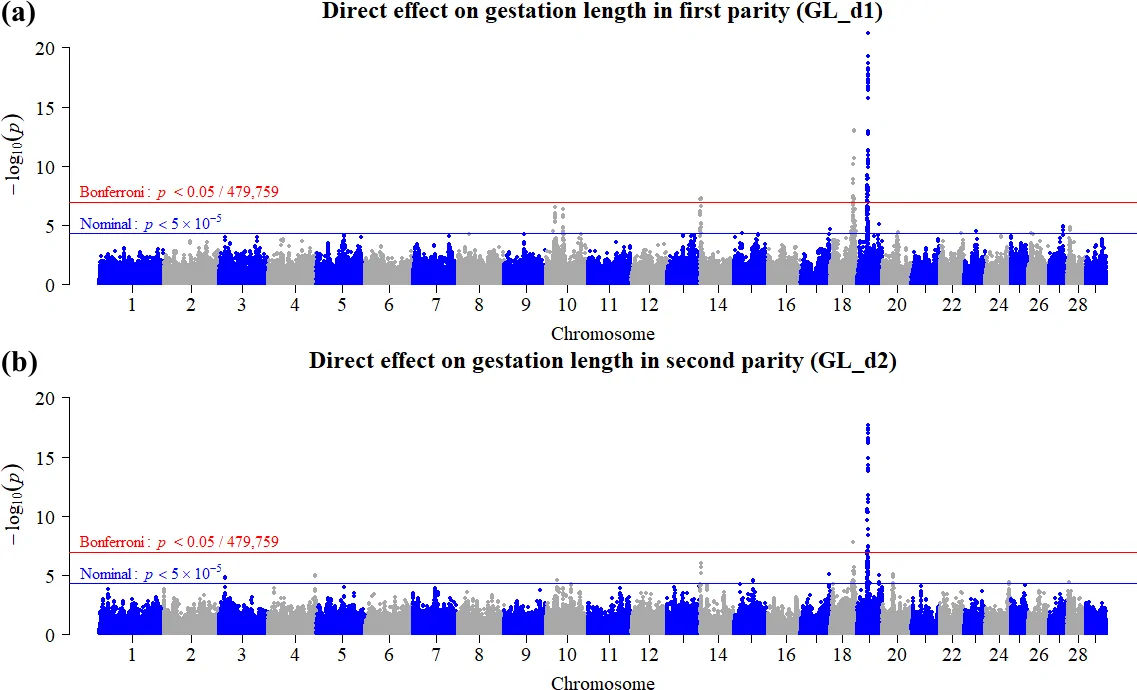
Manhattan plots from GWAS of gestation length (GL) for the direct effect in the first (GL_d1 – **a**) and second (GL_d2 – **b**) parity. The blue line indicates the nominal significance threshold ( $p\leq 5\times 10^{-5}$ ) and the red line the Bonferroni-corrected significance threshold ( $p\leq 1.04219\times 10^{-7}$ ).

**Figure 2:**
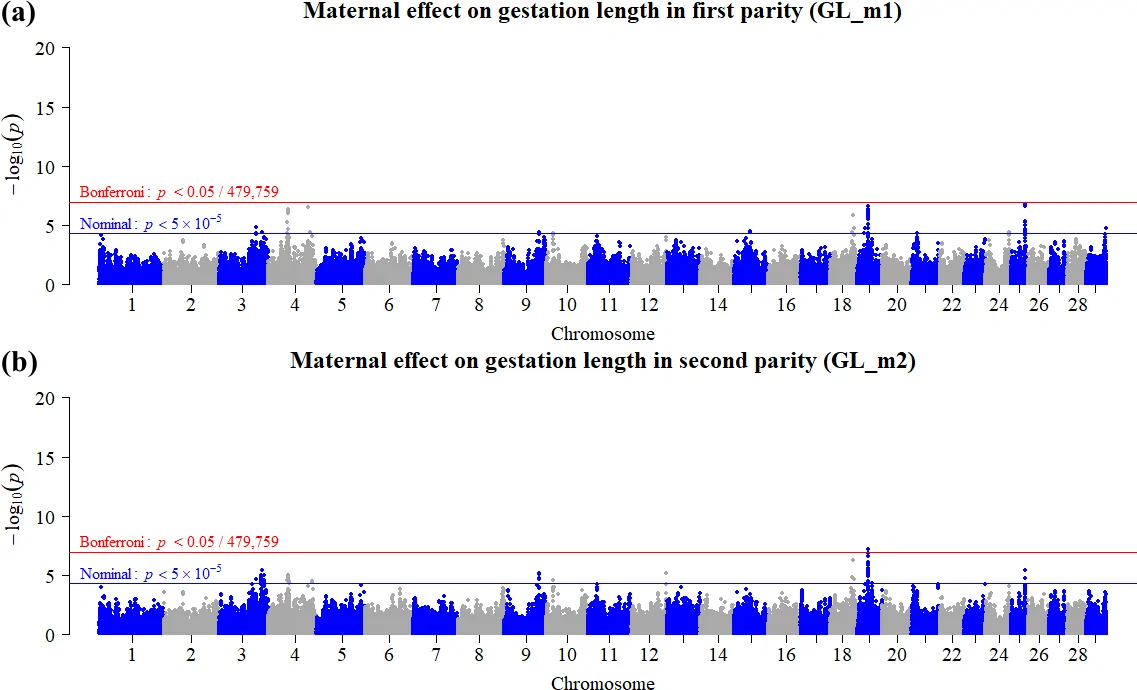
Manhattan plots from GWAS of gestation length (GL) for the maternal effect in first (GL_m1 – **a**) and second (GL_m2 – **b**) parity. The blue line indicates nominal significance threshold ( $p\leq 5\times 10^{-5}$ ) and the red line the Bonferroni-corrected significance threshold ( $p\leq 1.04219\times 10^{-7}$ ).

Table 1 contains the Cattle QTL Database entries within the 25 kb interval around significant SNPs for GL_d1 and GL_d2 in the category reproduction. The highest number was reported for QTLs for calving ease, SB, and age at first calving. In addition, five previously reported QTLs for GL were adjacent to variants associated with GL_d1. Consistent with the higher number of SNPs associated with GL_d1, significantly more QTLs were in proximity to SNPs associated with GL_d1 than to those with GL_d2. For all QTLs, the initial study, genomic position, and flanking markers are provided in Table S2 in the Supplement (Zölch et al., 2026b).

**Table 1:** Counts of Cattle-QTL-Database-listed reproduction QTLs found within ±25 kb of Bonferroni-significant GWAS variants for the direct effect on gestation length (GL) in the first (GL_d1) and second (GL_d2) parity.

Trait	QTL count	
	GL_d1	GL_d2
Age at first calving	7	6
Age at puberty	1	1
Birth index	2	0
Calf size	4	0
Calving ability	5	0
Calving ease	27	6
Conception rate	3	1
Gestation length	5	0
Pregnancy rate	2	0
Stillbirth	16	2

## 4 Discussion

The GWAS results point out several significant genomic regions affecting GL in German Holsteins, underlining the complex genetic architecture of GL. Notably, all SNPs were associated with direct effects on GL, except for one that was associated with GL_m2. This finding aligns with heritabilities estimated by Haile-Mariam and Pryce (2019). Their heritability estimates of GL direct were 0.28 in heifers and 0.36 in cows but only 0.04 for maternal GL. When considered together, this indicates a higher genetic effect of the calf on GL than of the dam.

More SNPs were significantly associated with GL_d1 than with GL_d2. The opposite might be expected, based on the higher heritability of GL_d2. Comparing their correlation to SB, only GL_d1 showed a significant moderate negative correlation (Zölch et al., 2026a). The most probable contributor to this difference is the sample size. The number of available DRPs for GL_d2 was more than 50 % smaller compared to GL_d1. If a larger dataset of second-parity cows becomes available in the future, it may be possible to determine whether a genomic difference between GL_d1 and GL_d2 exists.

The applied mixed linear model-based association analysis (MLMA) is rather conservative, as the SNP effect is modeled as both a fixed and a random effect (Yang et al., 2014). Although this can be mitigated by excluding the chromosome of the SNP tested for association from the GRM, it often requires the inclusion of additional principal components to adequately account for population structure (Schneider et al., 2024). As the genomic inflation factors in our analysis were close to 1 across all traits, suggesting little to no genomic inflation or deflation, we consider MLMA as suitable for GWAS of GL.

The validity of the identified genomic regions is further supported by the QTL mapping, which places them in the context of previous studies. The search for QTL within ±25 kb around genome-wide significant SNPs was justified by the results of Qanbari et al. (2010), who reported the highest linkage disequilibrium for German Holsteins in this range. Fang et al. (2019) have already reported a QTL on BTA14 affecting GL, and Maltecca et al. (2011) found two QTLs on BTA18 and BTA19. The SNP rs109478645 (BTA18:57137302), originally reported by Cole et al. (2009), showed the strongest association to direct GL in the analysis of Maltecca et al. (2011). This SNP was not part of our HD genotypes, but rs136803819 (BTA18: 57138190), which was significant for GL_d1 in our study, is located near to it (888 bp). Both SNPs are within the LOC618463 gene (BTA18:57124819–57141257), coding for sialic acid-binding Ig-like lectin 5 (SIGLEC5). Cole et al. (2009) proposed that elevated SIGLEC5 reduces the pool of free leptin, which in turn contributes to increased GL. This hypothesis is consistent with Puckowska et al. (2019), who reported an effect of SIGLEC5 on calving interval and with human data showing leptin's influence on gestation (Branham et al., 2022).

In addition, known QTLs for calving ease on BTA18 and BTA19 are in proximity to our GWAS results (Sahana et al., 2011; Abo-Ismail et al., 2017; Müller et al., 2017). Similarly, QTLs for SB are located on BTA18 and BTA19 – close to significant SNPs (Sahana et al., 2011; Abo-Ismail et al., 2017; Wu et al., 2017; Jakimowicz et al., 2022). BTA18 also harbors QTLs for calving ability (Abo-Ismail et al., 2017), pregnancy rate (Parker Gaddis et al., 2016), calf size, and birth index (Sahana et al., 2011). Found on BTA19 are QTLs for age at first calving (Prakapenka et al., 2023). The adjacent QTLs show that genomic regions significantly associated with GL frequently overlap with loci influencing other fertility traits, such as SB and calving ease. Currently, GL is not part of the routine breeding value estimation. Based on its relevance for fertility traits, especially the direct effect on GL, this should be implemented into genomic selection. Existing literature and our GWAS results can provide the starting point for the genetic improvement of GL.

## 5 Conclusions

In conclusion, we were able to identify GL-associated SNPs and link them to adjacent, previously published QTLs. In addition to GL, these QTLs also affect other calving traits, such as SB and calving ease. This relationship among traits highlights the importance of GL for fertility and supports our recent findings on local genetic correlations between GL and SB. Furthermore, the detected GL-associated genomic regions could be used for genomic selection and to filter results, e.g., in subsequent gene expression analyses. For this purpose, the focus should be on the direct effect on GL, as a higher genetic potential of change is to be expected.

## Supplement

10.5194/aab-69-563-2026-supplement The supplement related to this article is available online at https://doi.org/10.5194/aab-69-563-2026-supplement.

## Data Availability

The data are the property of the breeding organization and therefore cannot be made available to the public.
