# Supplementary material for: Genome-wide association study of gestation length in German Holsteins
Source: Arch Anim Breed. 2026 Oct 6;69(4):563–8. doi: 10.5194/aab-69-563-2026 (PMC13640962; doi:10.5194/aab-69-563-2026)
Supplement: Supplementary file 1 [file aab-69-563-2026-supplement.zip › aab-69-563-2026-supplement-title-page.pdf]

Supplement of Arch. Anim. Breed., 69, 563–568, 2026
https://doi.org/10.5194/aab-69-563-2026-supplement
© Author(s) 2026. CC BY 4.0 License.

Open Access
Archives Animal Breeding

Supplement of

Genome-wide association study of gestation length in German Holsteins

Maximilian Zölch et al.

Correspondence to: Maximilian Zölch (maximilian.zoelch@uni-hohenheim.de)

- aab-69-563-2026-supplement-title-page.pdf
- Table_S1.xlsx
- Table_S2.xlsx

The copyright of individual parts of the supplement might differ from the article licence.
